# Supplementary material for: Persistence of plant-mediated microbial soil legacy effects in soil and inside roots
Source: Nat Commun. 2021 Sep 28;12:5686. doi: 10.1038/s41467-021-25971-z (PMC8478921; doi:10.1038/s41467-021-25971-z)
Supplement: Supplementary file 1 — Supplementary Information [file 41467_2021_25971_MOESM1_ESM.pdf]

Supplementary Information for Persistence of plant-mediated microbial soil legacy effects in soil and inside roots

Authors

S. Emilia Hannula [1#*], Robin Heinen[1,2#], Martine Huberty[1,3], Katja Steinauer[1], Jonathan R. De Long[1,4], Renske Jongen[1], & T. Martijn Bezemer[1,3]

Supplementary Figure 1. The fungal community structure per previous and current plant species in all soils and across time.
Supplementary Figure 2. The bacterial community structure per previous and current plant species in all soils and across time
Supplementary Figure 3. The fungal community structure per plant species in all soils
Supplementary Figure 4. The resemblance of root bacterial and fungal community to soil communities measured
Supplementary Figure 5. Fungal and bacterial endophytes affected by current and previous plants
Supplementary Figure 6. Fungal classes and bacterial phyla related to plant performance
Supplementary Figure 7. Bacterial community structure affecting below-ground biomass per individual plant species.
Supplementary Figure 8. Fungal community structure affected by growing in own soils and in away soils at different times
Supplementary Figure 9. Bacterial community structure affected by growing in own soils and in away soils at different times.
Supplementary Figure 10. Potential plant pathogens and AMF in own soils and in soils of plants from other species and in grass and forb soils in time
Supplementary Figure 11. Fungi affected by growing in own soil.
Supplementary Figure 12. Bacteria affected by growing in own soil.
Supplementary Figure 13. Bacteria and fungi affected in species-specific manner for growing in own soil
Supplementary table 1. Primers used in this study.

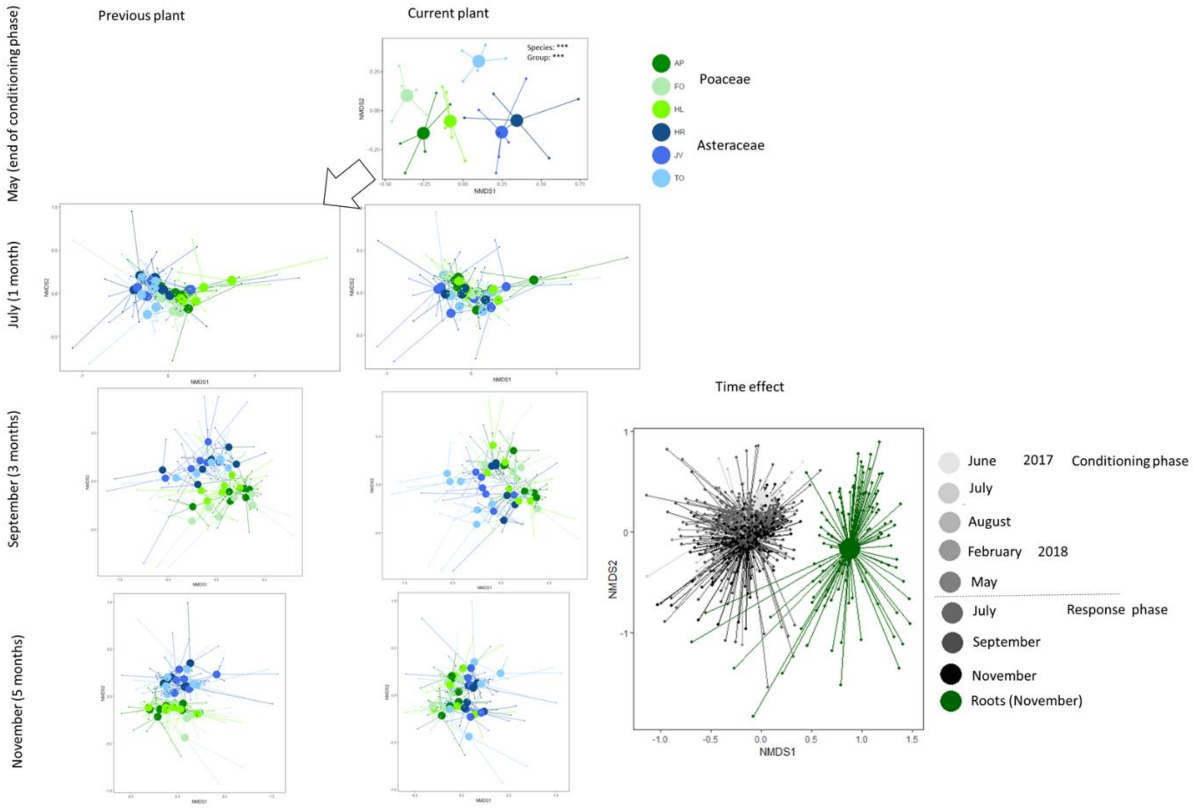

Supplementary figure 1. The fungal community structure per previous and current plant species in all soils and across time. The composition of fungal community at the end of the conditioning phase, and 1, 3 and 5 months after the start of the experiment depicted as centroids with variance (5 replicate containers) for each plant species with NMDS based on Bray Curtis dissimilarity. The mesocosms are colored based on conditioning (previous) plant species and by current plant species. The samples from the beginning are based on conditioning with current plant for 12 months and become the ‘previous plant’ for the new containers. The times are organized from light (June 2017; conditioning phase) to darker (November 2018; response phase) and contain data from the conditioning phase of the experiment (published earlier in Hannula et al. 2019) for comparison purposes. Root samples are marked in time series as green. Note that DNA from roots were extracted using different extraction kit and are hence not fully comparable. The 2D stress values for end of conditioning phase is 0.18, for 1 month is 0.15, for 3 months 0.12 and for 5 months 0.14, and for the all data presenting variation in time 0.19.

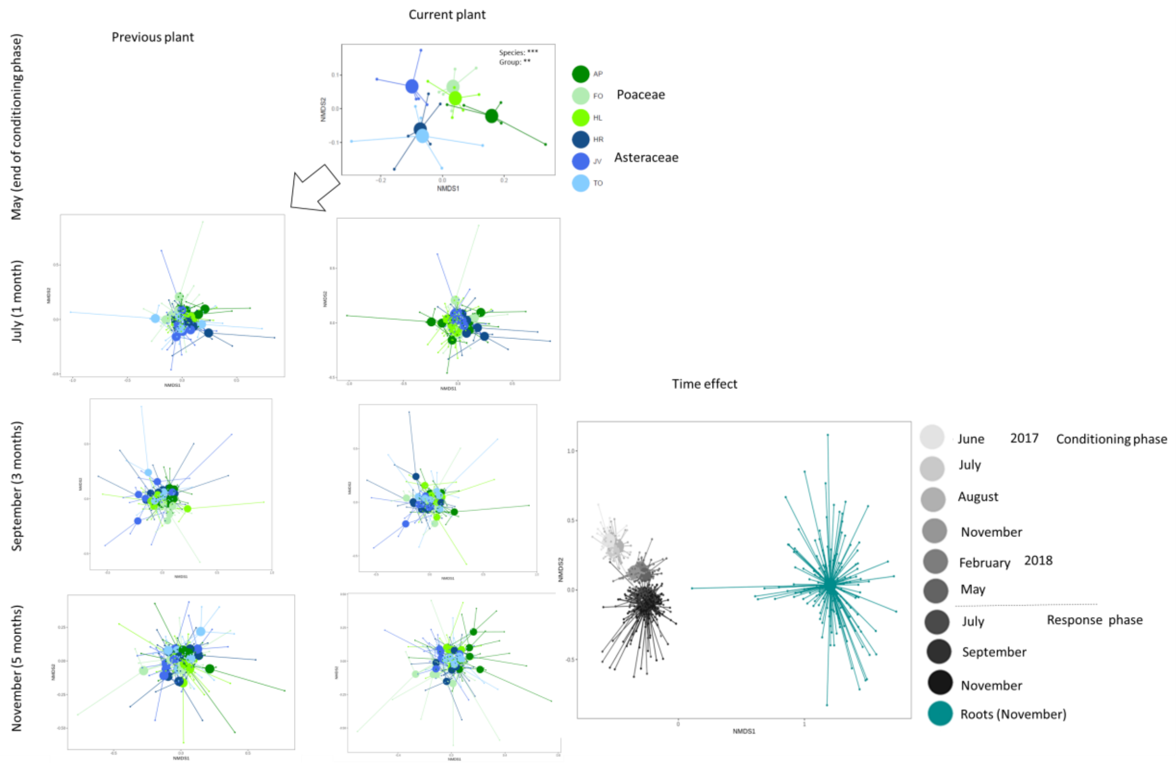

Supplementary figure 2. The bacterial community structure per previous and current plant species in all soils and across time. The composition of bacterial community at the end of the conditioning phase, and 1, 3, and 5 months after the start of the experiment depicted as centroids with variance (5 replicate containers) for each plant species with NMDS based on Bray Curtis dissimilarity. The mesocosms are colored based on conditioning (previous) plant species and by current plant species. The samples from the beginning are based on conditioning with current plant for 12 months and become the 'previous plant' for the new containers. The times are organized from light (June 2017; conditioning phase) to darker (November 2018; response phase) and contain data from the conditioning phase of the experiment (published earlier in Hannula et al. 2019) for comparison purposes. Root samples are marked with turquoise. Note that DNA from roots were extracted using different extraction kit and are hence not fully comparable. The 2D stress values for end of conditioning phase is 0.14, for 1 month is 0.18, for 3 months 0.17 and for 5 months 0.15. The 2D stress value for the full time comparison was 0.21.

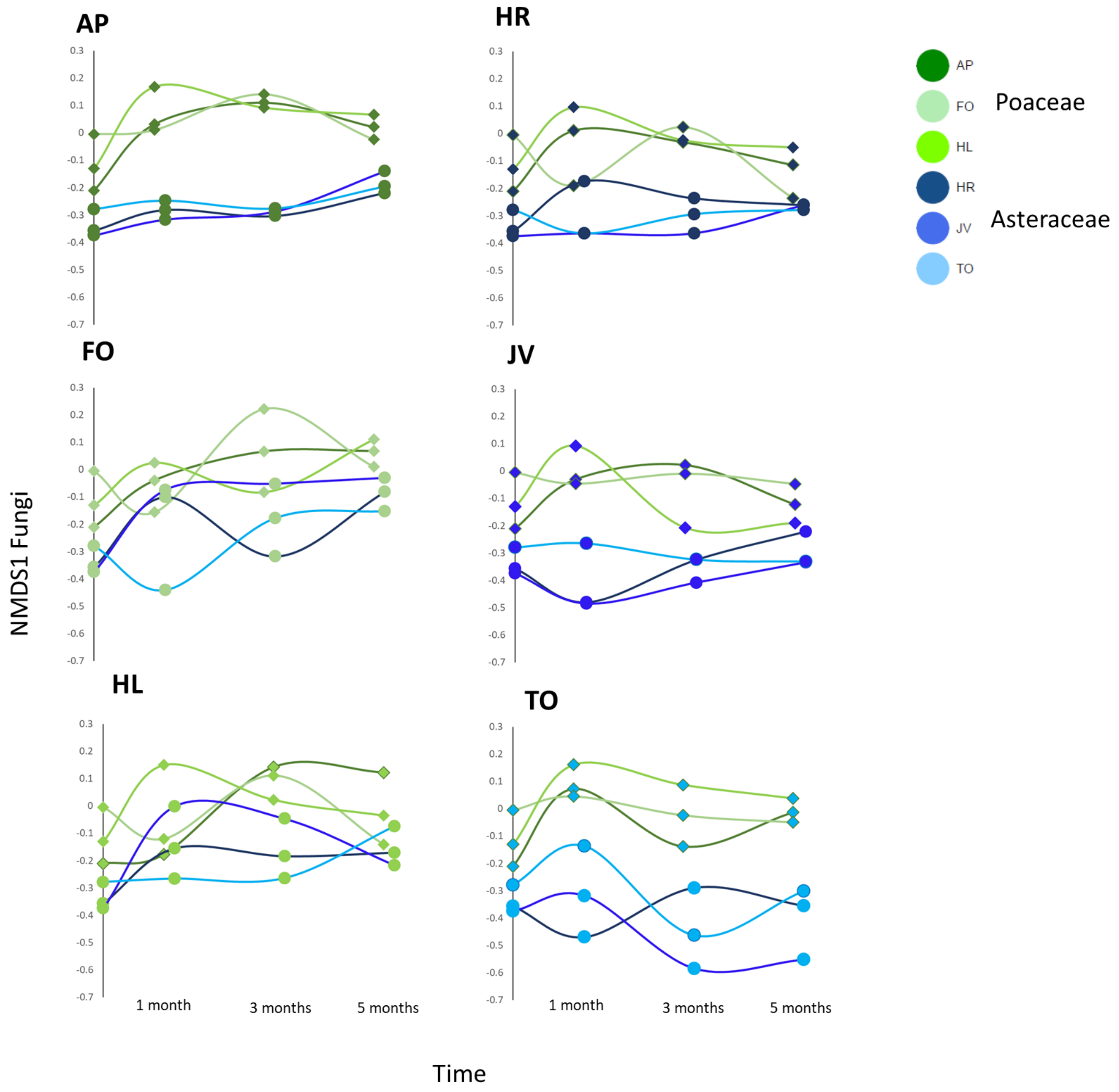

Supplementary figure 3. The fungal community structure per plant species in all soils. NMDS1 axis of fungal community per plant species (marker colors) and per soil types (line colors) as function of time. The NMDS is based on Bray Curtis dissimilarity and full data with variation is shown in Figure S1.

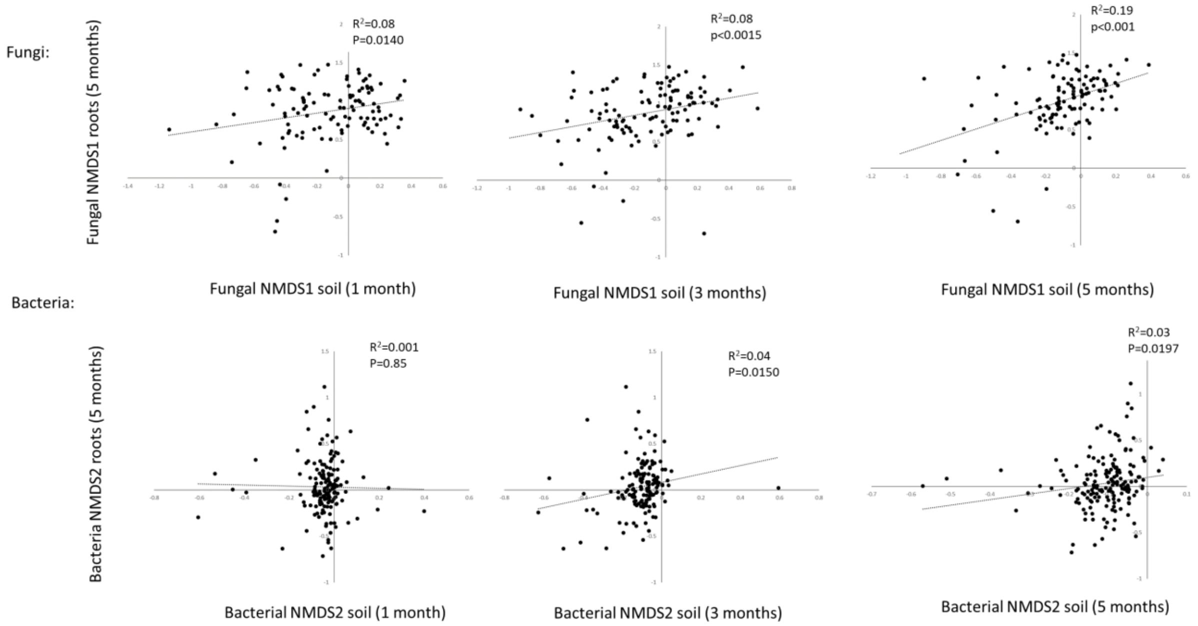

Supplementary figure 4. The resemblance of fungi and bacteria inside roots measured with NMDS1 (fungi) and NMDS2 (bacteria) to fungi and bacterial communities in the soil measured at different time points. Pearson correlation coefficient and statistical significance are indicated for each time point.

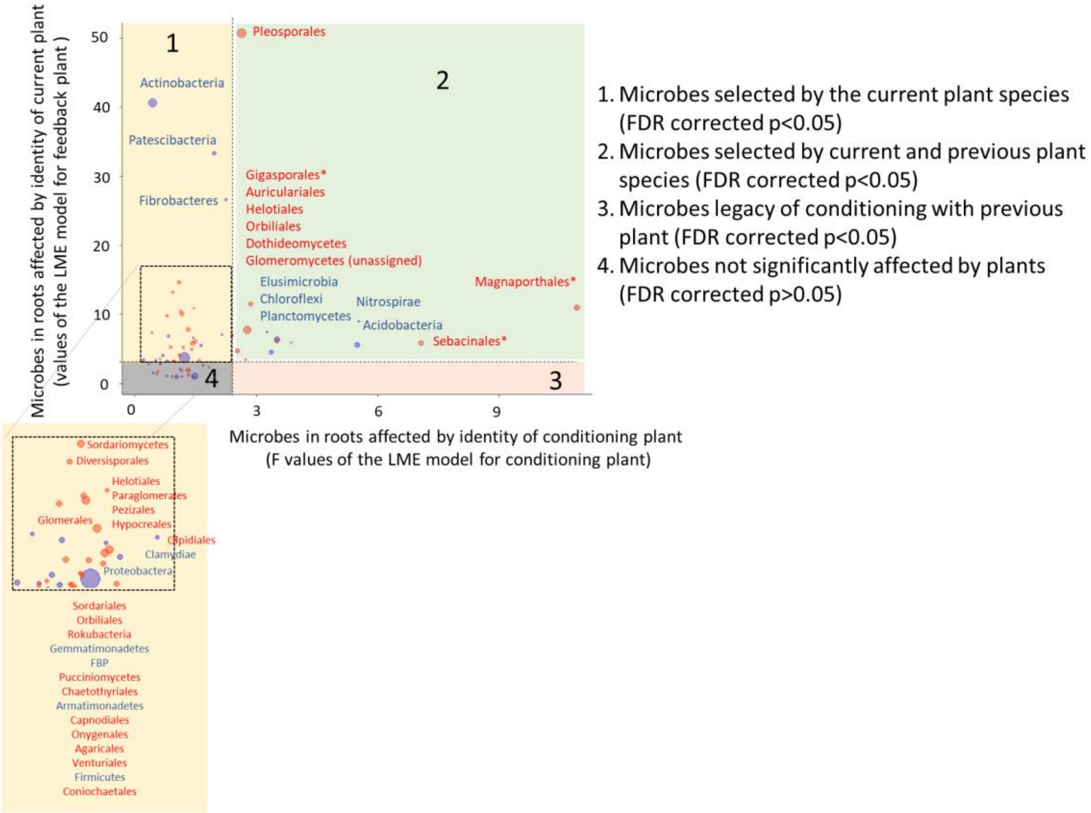

Supplementary figure 5. Endophytic fungal orders (red) and bacterial phyla (blue) whose abundances are significantly (FDR $p<0.05$) changed by conditioning plant species (3, orange), responding plant species (1, yellow) and by both (2, green). The dashed lines indicate the minimum F values that are significantly explaining the LME model after FDR correction.

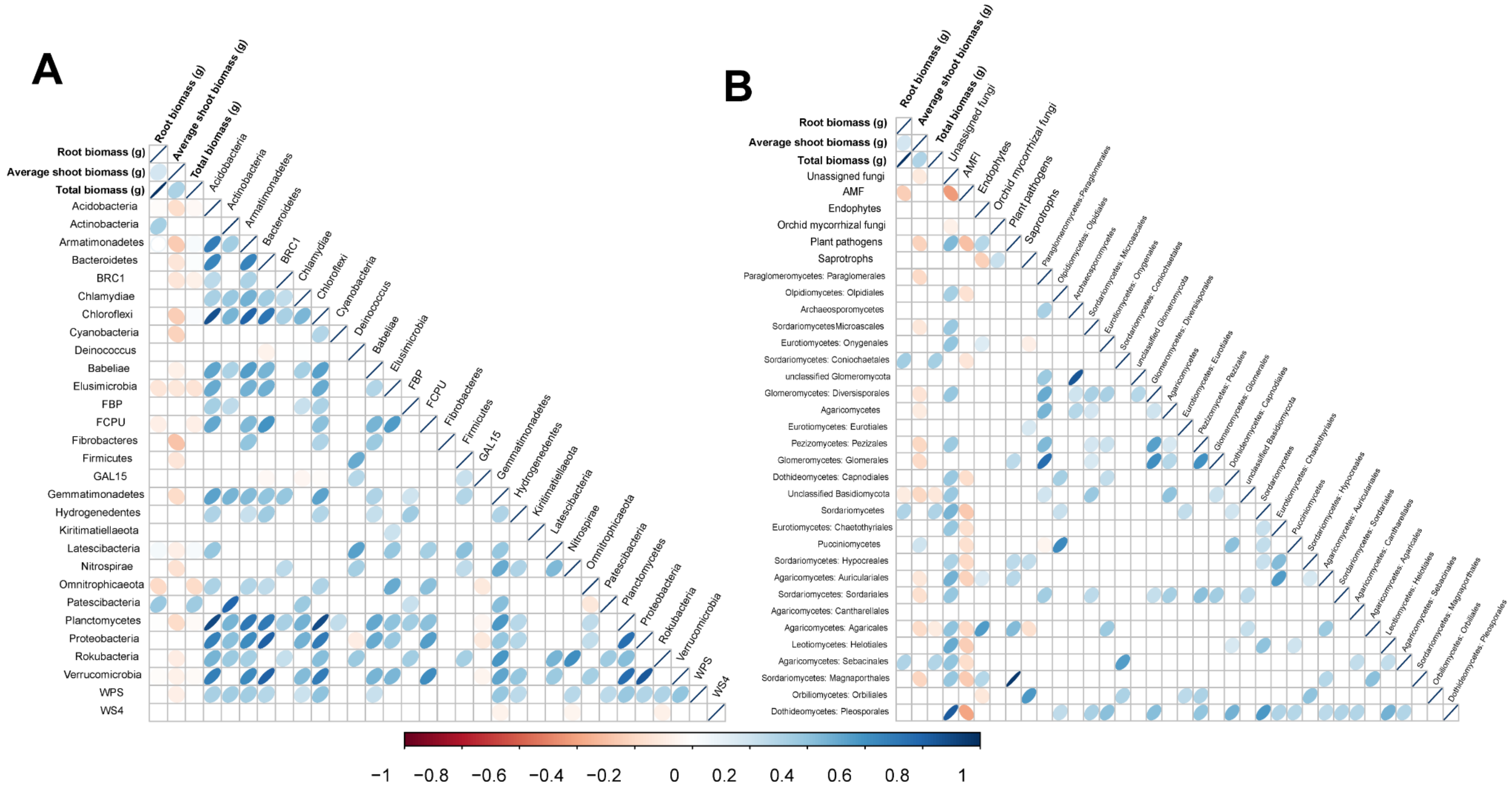

Supplementary figure 6. Correlation plot of relationship between endophytic bacterial phyla (A) and fungal functional guilds and classes (B) with root and shoot biomass of current plants. Only significant correlations after correction for multiple correlations (FDR correction; $p<0.01$; ref 75) are shown. The direction of correlations is marked so that significant negative correlations are marked in red and positive significant correlations with blue.

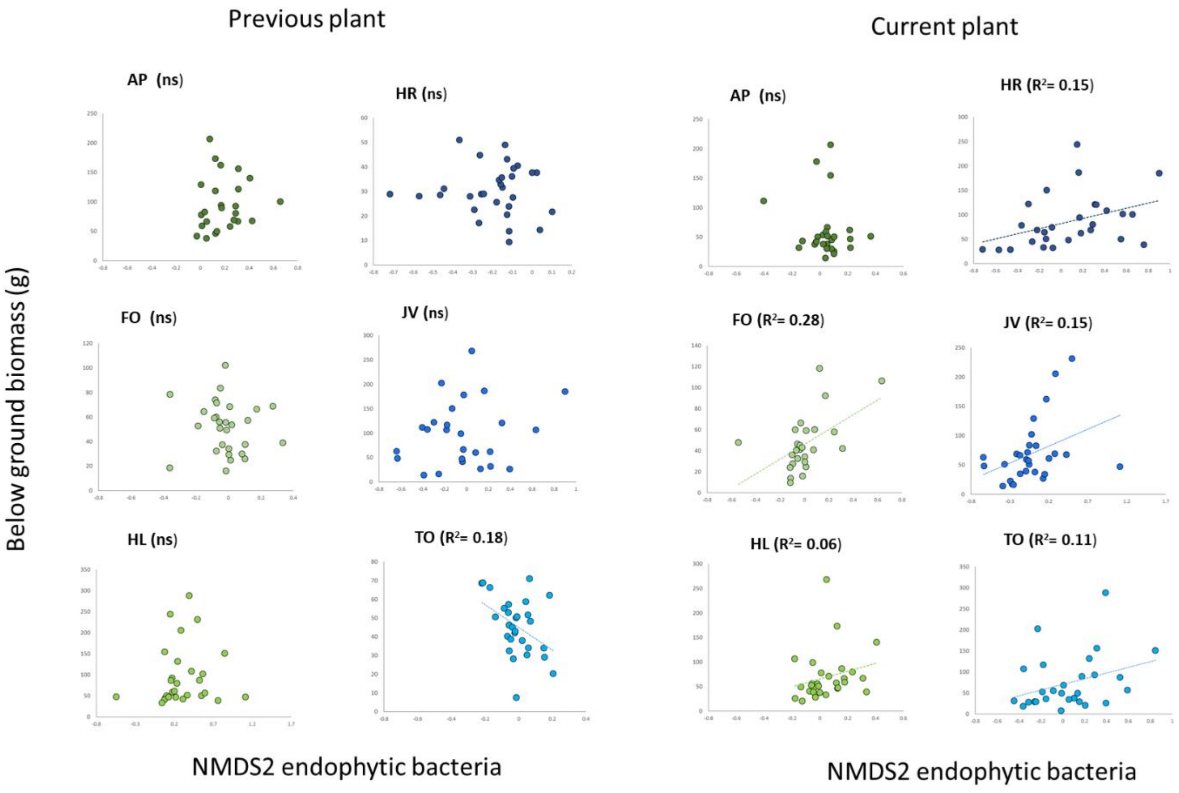

Supplementary figure 7. Relationship between endophytic bacterial community structure measured with NMDS2 and belowground plant biomass divided per previous soil and current plant. Only for significant correlations, the correlation coefficient is shown.

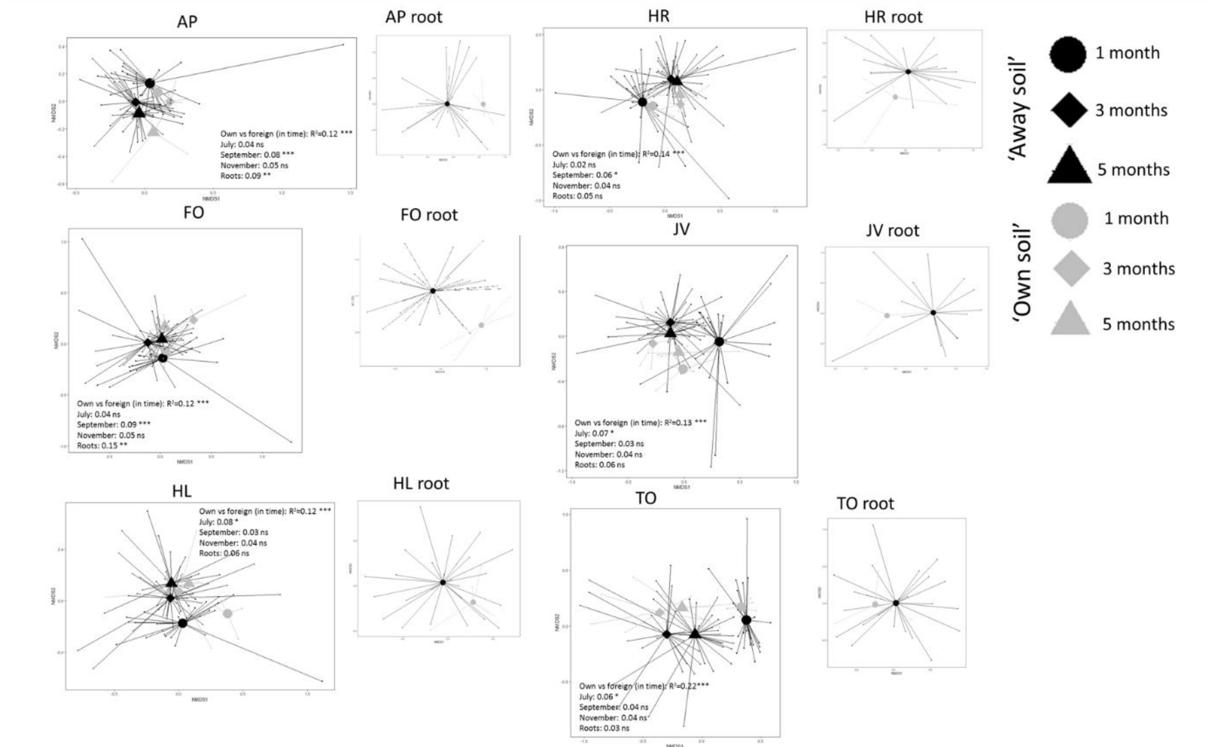

Supplementary figure 8. Fungal community structure affected by growing in own soils and in away soils at different times. Fungal communities in own (grey) and other soils from other plants (black) in time (symbols) per plant species. The fungal communities inside the roots are separately depicted. Statistical significance from PerMANOVA is given in the figure.

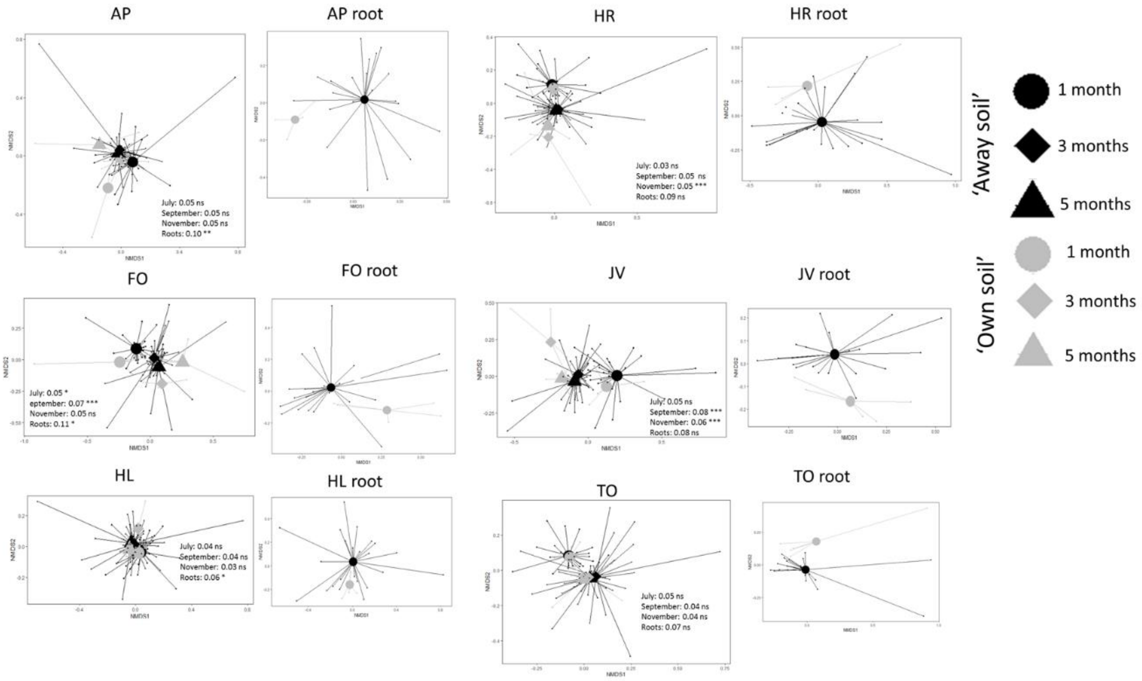

Supplementary figure 9. Bacterial community structure affected by growing in own soils and in away soils at different times. Bacterial communities in own (grey) and other soils from other plants (black) in time (symbols) per plant species. The bacterial communities inside the roots are separately depicted. Statistical significance from PerMANOVA is given in the figure.

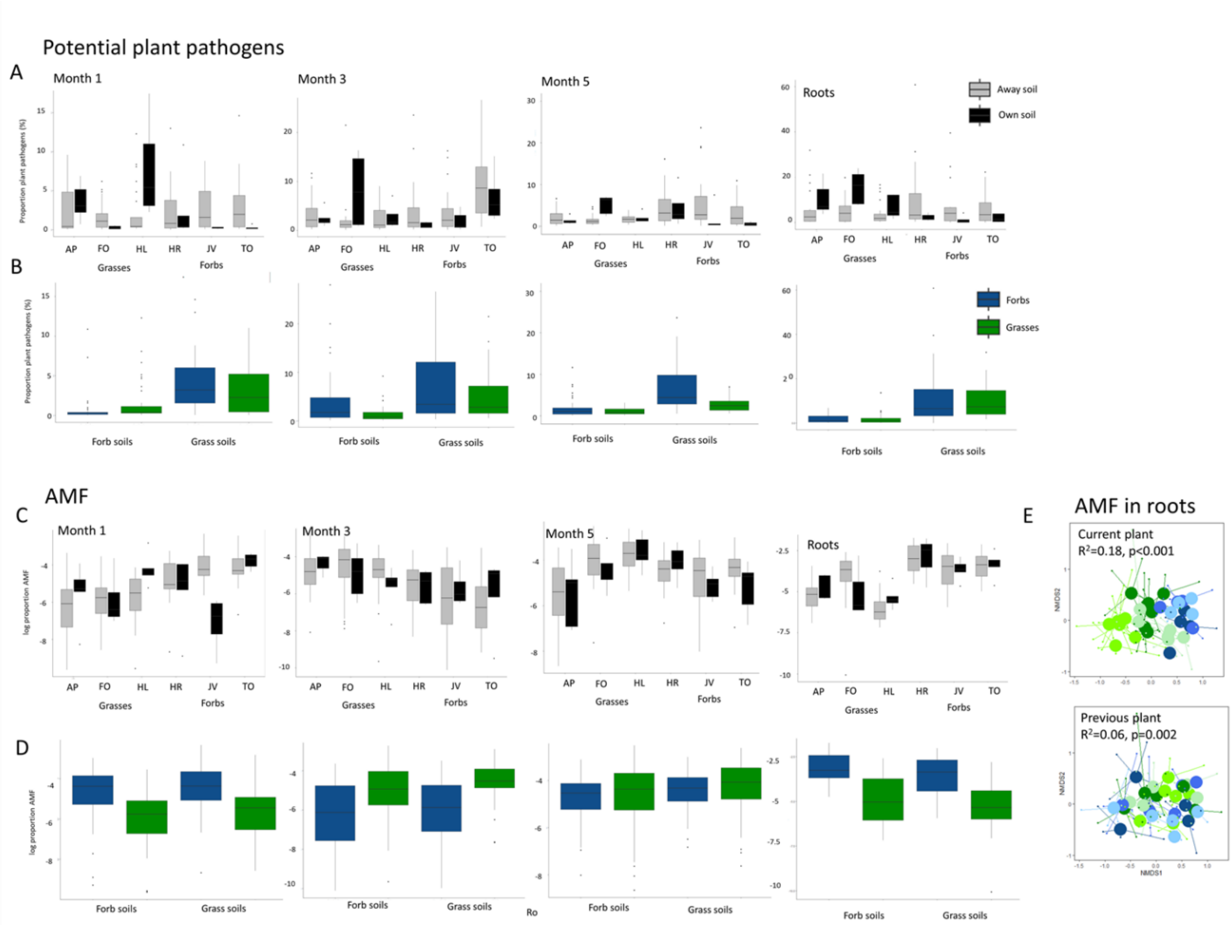

Supplementary figure 10. Potential plant pathogens and AMF in own soils and in soils of plants from other species and in grass and forb soils in time. Relative abundance of potential plant pathogens (A & B) and AMF (C & D) for all plants and time points when grown (A & C) in own soil (black) or away soils (grey) and plant functional groups grown in the soil of the same functional group or in the soil of another functional group. Blue colors denote forbs and green color grasses. The Tukey box-and-whisker-plots depict median percentage of plant pathogens (A&B) and AMF (C&D) in either own vs. away soils in each block (n=5; A&B) or between plants conditioned with plants from different plant families (n=30; C&D). (E) The community structure of AMF inside the roots based on Bray-Curtis distance of all fungi classified as ‘Glomeromycota’ colored by current plant and pervious plant. The 2D stress value of the NMDS plots is 0.16.

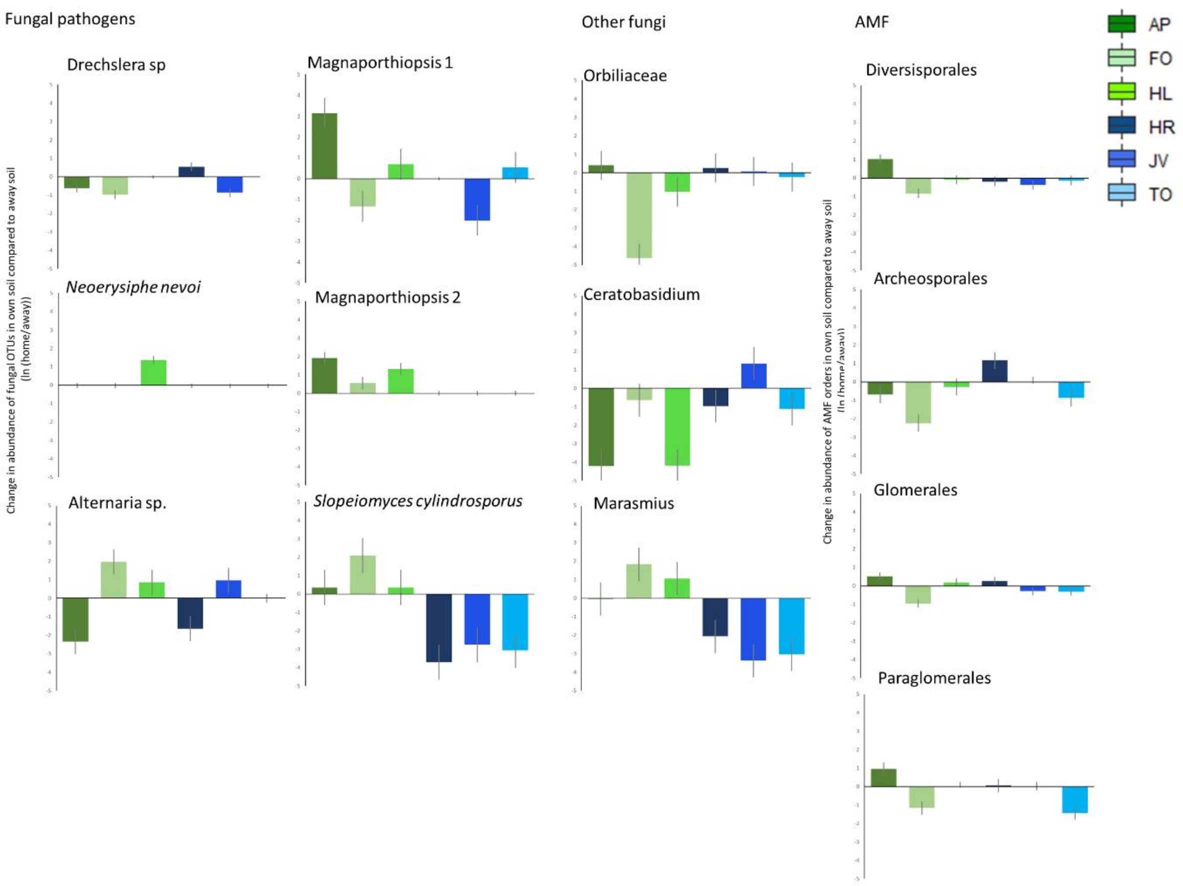

Supplementary figure 11. Fungi enriched or decreasing when plants grow in their own soil. Phylotypes of fungal plant pathogens, and other fungi and orders of AMF inside the roots affected significantly by plants growing in their own soil compared to growing in another soil calculated using formula ln(home/away). Average relative abundance of each taxa and standard error between replicate blocks (n=5) are shown. Blue colors denote forbs and green colors grasses.

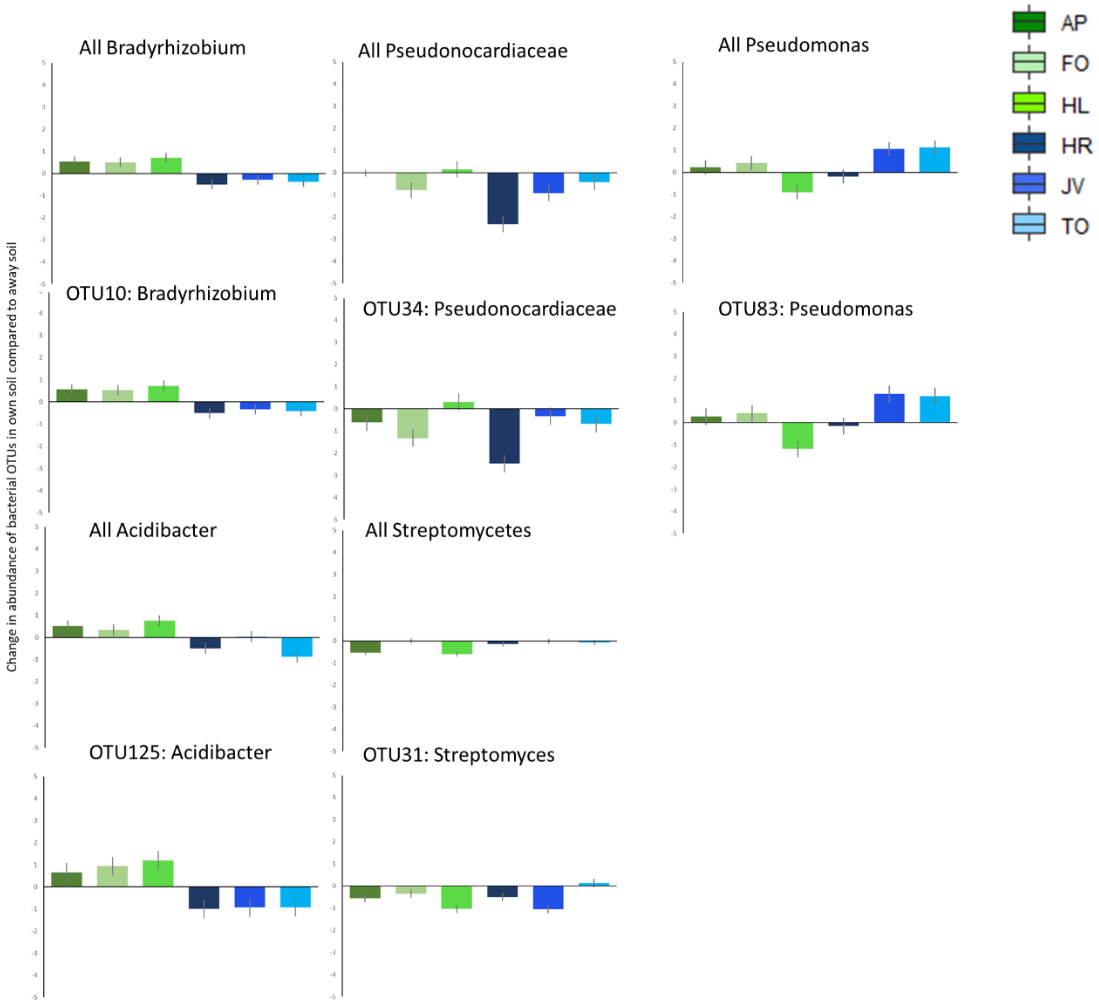

Supplementary figure 12. Bacteria enriched or decreasing when plants grow in their own soil. Bacterial species and genera inside the roots affected significantly by plants growing in their own soil compared to growing in another soil calculated using formula ln(home/away). Average relative abundance of each taxa and standard error between replicate blocks (n=5) are shown. Blue colors denote forbs and green colors grasses.

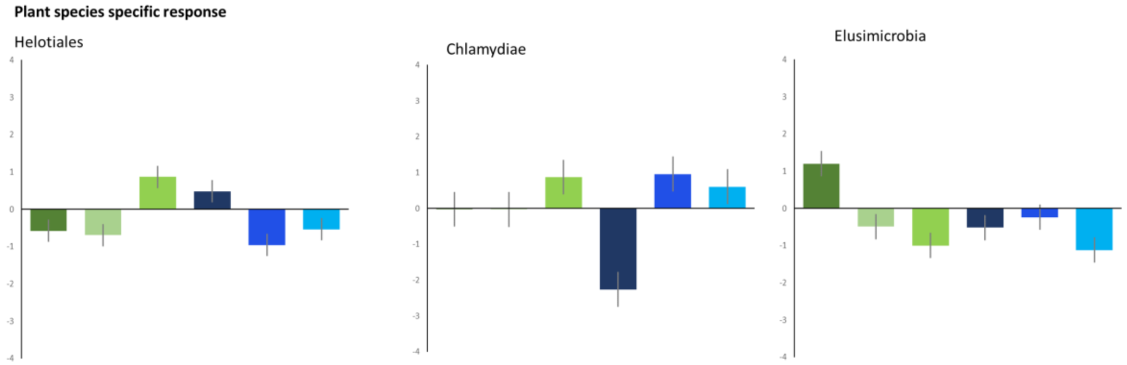

Supplementary figure 13. Fungal class and bacteria phyla enriched or decreasing when plants grow in their own soil in a species-specific manner calculated using formula ln(home/away). Average relative abundance of each taxa and standard error between replicate blocks (n=5) are shown. Blue colors denote forbs and green colors grasses.

Notes: Species specific effects on endophytic fungi and bacteria: Species-specific responses to own and foreign soil were detected for Chlamydiae (interaction own-soil*plant species LME: $F=4.18$, $p<0.001$) and Elusimicrobia (interaction own-soil*plant species LME: $F=5.10$, $p<0.001$). The relative abundance of the fungal class Helotiales was affected when grown in own soil, but in a plant species-specific manner (LME: $F=2.95$, $p=0.014$, Fig. S12). For Helotiales, most sequences could not be assigned further than as '*Helotiales* sp.' and only three species that were present in more than 20 samples could be identified in more detail. The family responsible for the increase in relative abundance of Helotiales in *H. lanatus* soils was Hyaloscyphaceae that was only present in the roots if *H. lanatus* grew in its own soil.

Supplementary table 1. Primers used in this study. The ITS3NGS1-ITS3NGS5 primers were mixed in equal proportion to ensure amplification of all fungal groups (62).

Name	Target	Sequence	Reference
ITS4NGS	Fungi (reverse)	TCCTSCGCTTATTGATATGC	62
ITS3NGS1	Fungi (forward mixture)	CTAGACTCGTCATCGATGAAGAACGCAG	62
ITS3NGS2	Fungi (forward mixture)	CTAGACTCGTCAACGATGAAGAACGCAG	62
ITS3NGS3	Fungi (forward mixture)	CTAGACTCGTCACCGATGAAGAACGCAG	62
ITS3NGS4	Fungi (forward mixture)	CTAGACTCGTCATCGATGAAGAACGTAG	62
ITS3NGS5	Fungi (forward mixture)	CTAGACTCGTCATCGATGAAGAACGTGG	62
515FB	Bacteria (forward)	GTGYCAGCMGCCGCGGTAA	63;64
806RB	Bacteria (reverse)	GGACTACNVGGGTWTCTAAT	63;65
