# Supplementary material for: Persistence of plant-mediated microbial soil legacy effects in soil and inside roots
Source: Nat Commun. 2021 Sep 28;12:5686. doi: 10.1038/s41467-021-25971-z (PMC8478921; doi:10.1038/s41467-021-25971-z)
Supplement: Supplementary file 3 — Description of Additional Supplementary Information [file 41467_2021_25971_MOESM3_ESM.pdf]

Description of Additional Supplementary Files

File Name: Supplementary Movie 1

Description: How mesocosms were divided into smaller mesocosms
